# Plant Growth Regulators Use in the In Vitro Culture of *Agave* Species

**DOI:** 10.3390/plants14213402

**Published:** 2025-11-06

**Authors:** Estefany Alejandra Sánchez-Mendoza, Eugenio Pérez-Molphe-Balch, Rafael Guzmán-Mendoza, Graciela Ruiz-Aguilar, Alberto M. García-Munguía, Rogelio Costilla-Salazar, Héctor Gordon Núñez-Palenius

**Affiliations:** 1 División de Ciencias de la Vida, Campus Irapuato-Salamanca, Universidad de Guanajuato, Irapuato 36500, Mexico; 2 Unidad de Biotecnología Vegetal, Centro de Ciencias Básicas, Universidad Autónoma de Aguascalientes, Aguascalientes 20131, Mexico; 3 Departamento de Agronomía, División de Ciencias de la Vida, Campus Irapuato-Salamanca, Universidad de Guanajuato, Irapuato 36500, Mexico; 4 Departamento de Ciencias Ambientales, División de Ciencias de la Vida, Campus Irapuato-Salamanca, Universidad de Guanajuato, Irapuato 36500, Mexico; 5 Laboratorio de Parasitología Agrícola, Centro de Ciencias Agropecuarias, Universidad Autónoma de Aguascalientes, Jesús María 20900, Mexico

**Keywords:** auxins, *Agave* spp., cytokinins, micropropagation, organogenesis, plant growth regulators (PGRs), polyamines, somatic embryogenesis, culture in vitro

## Abstract

*Agave* species possess substantial cultural, ecological, and economic significance, particularly in Mexico, where they are traditionally utilized for food, fiber, and beverages. Their industrial relevance has expanded to include bioenergy, nutraceuticals, and sustainable agriculture. However, conventional propagation methods are constrained by long life cycles, low seed germination rates, and susceptibility to phytopathogens. In vitro culture has emerged as a pivotal biotechnological strategy for clonal propagation, germplasm conservation, and physiological enhancement. This review presents a critical synthesis of plant growth regulators (PGRs) employed in agave micropropagation, emphasizing their roles in organogenesis, somatic embryogenesis, shoot proliferation, and rooting. Classical PGRs such as 6-benzylaminopurine (BAP), benzyladenine (BA), 2,4-dichlorophenoxyacetic acid (2,4-D), indole-3-acetic acid (IAA), and indole-3-butyric acid (IBA) are widely utilized, with BA + 2,4-D and BA + IAA combinations demonstrating high efficiency in embryogenic callus induction and shoot multiplication. Additionally, non-traditional regulators such as abscisic acid (ABA) and putrescine (Put) have been shown to affect embryo maturation. This review synthesizes recent studies on agave in vitro culture protocols, identifies trends in PGR use, and highlights key research gaps. These insights reveal opportunities for innovation and underscore the need for species-specific optimization and molecular validation to improve reproducibility and scalability.

## 1. Introduction

The *Agave* genus represents a plant resource of exceptional cultural, ecological, and economic importance, with Mexico recognized as its primary center of origin and diversification [1,2]. Historically, *Agave* species have been widely used in the production of fibers, food, and fermented beverages [3,4,5]. In recent decades, their applications have expanded to include the production of syrups, prebiotics, livestock feed, by-product valorization, and biofuels [6,7,8,9]. This versatility has driven increasing scientific interest in optimizing the cultivation and propagation of *Agave* species, particularly in response to industrial demand and the urgent need for biodiversity conservation [10].

Plant tissue culture is a fundamental biotechnological tool, as the ability to precisely control the physicochemical conditions of the culture environment has enabled its application in clonal propagation, genetic conservation, and physiological improvement of various plant species [11,12,13]. Furthermore, this technique has proven effective for generating cell suspensions, protoplasts, and callus tissues, which represent viable biomass alternatives to produce secondary metabolites of industrial and pharmacological interest [14,15,16]. Likewise, tissue culture is employed as a strategy for conserving germplasm and plants with low reproductive capacity. By maintaining explants under controlled conditions and using media supplemented with growth regulators that inhibit development, it is possible to preserve genetic material in a reduced growth state, facilitating long-term storage and significantly reducing labor and maintenance costs [17].

In the case of agave, these methodologies offer a solution to overcome limitations associated with long life cycles, low germination rates, and reproductive constraints, thereby enabling more efficient and sustainable utilization [18,19,20,21]. Although other applications, such as cell suspensions or secondary metabolite production, remain limited in *Agave* species, in vitro propagation and conservation systems have been successfully implemented, relying primarily on the appropriate use of key factors that regulate cellular responses. Among these, PGRsplay a central role in modulating physiological, morphogenetic, and biochemical processes, thereby increasing the efficiency and reproducibility of established protocols [22,23]. These systems provide a controlled environment that facilitates the analysis of interactions between PGRs and genetic factors, which is essential for understanding plant physiology and developing optimized strategies for the propagation and conservation of *Agave* species [12].

This review highlights the broad diversity in the use of PGRs in tissue culture protocols applied to *Agave* species, aiming to evaluate their effectiveness in morphogenic induction, clonal propagation, and germplasm conservation. The compiled data demonstrate significant progress, with PGRs playing a central role in the development of micropropagation strategies, primarily through direct and indirect organogenesis, as well as somatic embryogenesis.

Among the most frequently used PGRs are benzyladenine (BA), 2,4-dichlorophenoxyacetic acid (2,4-D), indole-3-acetic acid (IAA), and abscisic acid (ABA), applied in various combinations and concentrations [17]. BA stands out as the most versatile regulator, promoting organogenesis, somatic embryogenesis, and axillary shoot proliferation; however, its effectiveness depends on the type of explant and the species [13]. The morphogenic responses induced include direct and indirect organogenesis, somatic embryogenesis, callus formation, and rooting.

Given the predominance of auxins and cytokinins in current studies, the exploration of novel PGR groups such as brassinosteroids, polyamines, strigolactones, and signaling peptides remains scarce [23].

To support this analysis, the present review employed a structured methodological strategy that facilitated a systematic and critical examination of the relevant scientific literature. The research question was formulated using the SPIDER framework, proposed by Cooke, Smith, and Booth [24], designed to guide qualitative and thematic reviews. This framework comprises five components: S (Sample), referring to plant tissue culture in *Agave* species; Pi (Phenomenon of Interest), focused on the application of PGRs; D (Design), including experimental studies, scientific reviews, and academic book chapters addressing theoretical principles, protocols, and PGR-induced responses; E (Evaluation), involving the analysis of morphogenesis, shoot proliferation, root development, biomass accumulation, and physiological or biochemical changes; and R (Research Type), encompassing qualitative, descriptive, and experimental research. Additionally, the methodological approach proposed by Pardal-Refoyo and Pardal-Peláez [25] was adapted, encompassing stages such as formulating the research question, defining eligibility criteria, designing the methodological approach, conducting a database search, assessing bias, interpreting results, and updating the review. The integration of these frameworks enabled precise delimitation of search and selection criteria, facilitating the identification of trends, effects, limitations, and future perspectives regarding the use of PGRs in the tissue culture of *Agave* species, and providing a critical and up-to-date synthesis of the scientific evidence.

## 2. Historical, Economic, and Biotechnological Relevance of the *Agave* Species

The genus *Agave* belongs to the family Asparagaceae, subfamily Agavoideae [26], and is one of the most diverse plant genera in Mexico. Approximately 79% of *Agave* species are found in Mexico, with nearly 84% being endemic [1,27]. These species thrive across a wide range of habitats, including valleys, plains, rocky hillsides, and high-altitude mountainous regions, reflecting their broad geographical distribution within the country [28]. Agave is a plant of considerable cultural, ecological, and economic significance in many Mexican regions [29].

Historical knowledge of the *Agave* genus in Mexico dates back to pre-Hispanic times. Mesoamerican cultures extensively utilized various *Agave* species, integrating them into daily life, the economy, and regional cultural identity [29,30]. Among these, *Agave salmiana* L. and *Agave americana* L. were particularly valued for their roles in food, textiles, and ritual practices [2,10,31]. Approximately 70 traditional uses of agave have been documented, showcasing its continued importance from pre-Columbian times to the present day [32].

Among the earliest uses were fibers extracted from *Agavefourcroydes* Lem. and *Agave sisalana* Perr., used to produce baskets and textiles. Various species were also consumed as food, including their flowers, the inflorescence (known as “quiote”), and the basal stem (known as “piña”) [32]. One of the most culturally significant uses has been the production of fermented beverages. *A. salmiana* Otto ex Salm-Dyck and *A. mapisaga* Trel. are used to extract aguamiel, which, when fermented, yields pulque, a beverage with deep cultural roots and high nutritional value [33,34]. Following European colonization, distillation techniques were introduced, giving rise to spirits such as tequila, from *Agave tequilana* Weber, and mezcal, produced from various species, including *A. angustifolia* Haw., *A. potatorum* Zucc., *A. americana* var. *Oaxacensis* Gentry, *A. duranguensis* Gentry, and *A. cupreata* Trel. & Berger [35,36,37].

The cultivation of agave and the production of distilled spirits in Mexico represent not only a significant cultural heritage but also one of the most important activities in the agricultural and food sector [35]. Economically, Tequila ranks as the second highest export-generating product. In 2023, international tequila sales increased by 3.06% compared to the previous year [37].

Considering this, it is essential to promote the growth and diversification of the agave industry in Mexico to maintain global leadership. Although the country is currently a leader in the production of agave-derived spirits, other nations are beginning to explore this plant as a basis for new industries [35,36,37].

Beyond traditional uses, the potential of agave for bioproduct development continues to expand, particularly in the industrial, pharmaceutical, and energy sectors [38,39,40]. Its biochemical richness and adaptability make it a strategic crop for arid and semi-arid regions [10].

Several species have been identified as valuable sources of agavins (inulin from agave), a type of dietary fiber with prebiotic properties widely used as a nutritional supplement due to its digestive health benefits [41,42,43]. Moreover, industrial processing of agave generates substantial by-products and waste, including discarded leaves, bagasse, and vinasse [44]. These residues have drawn increasing attention for their potential as raw materials in the production of biofuels, fodder, and other high-value bioproducts [45,46].

Globally, countries such as Brazil have conducted studies on *Agave* species to produce dietary carbohydrates, fiber, and biofuels [9,47]. The use of agave as a biomass source for energy generation presents a strategic alternative for the energy transition, owing to its high biomass accumulation, physiological efficiency, and adaptation to semi-arid conditions [48]. Its non-competitive use of food crops further enhances its sustainability [49]. Brazil, the world’s largest producer of *A. sisalana* Perr. (sisal), presents an opportunity to valorize agave waste through anaerobic digestion processes, thereby contributing to bioenergy generation and reducing environmental impacts [50].

Among the most agriculturally important *Agave* species in Mexico is *A. tequilana* Weber, which has also attracted international attention as a sustainable, high value crop [37]. It is now considered a strategic species for the sustainable production of bioethanol, hydrogen, and distilled beverages due to its high biomass yield and low water requirements. In Australia, commercial cultivation is still in its early stages, with production limited to Queensland and pilot studies underway in other regions. In 2019, thirty hectares were planted, producing between 400 and 900 tons of biomass after five years. Its ability to grow in dry or semi-arid conditions with minimal inputs makes it a viable option for boosting rural economies [51,52]. Current global initiatives emphasize the need to transform the agave industry into a viable biomass-based sector [53].

Given the growing importance of *Agave* species and the rising demand for strategies to select improved genotypes and establish more productive plantations, optimizing propagation protocols is crucial. These protocols should ensure higher reproducibility, reduced costs, and enhanced plant resilience under stress conditions. This calls for the development of innovative techniques, including the use of elicitor compounds and strategies to improve specific physiological and morphological traits [13]. However, the physical structure of agave presents challenges for its manipulation under controlled conditions. Its large size, spiny leaves, and robust foliar architecture complicate handling [54].

In addition, its extended life cycle, which may exceed eight to twelve years before flowering, slows down breeding programs and genetic studies [13]. As a monocarpic species, agave flowers only once before entering senescence and dying [10].

These limitations underscore the need to develop innovative strategies to manipulate growth and development and facilitate plant material production. In this regard, in vitro culture emerges as a potential approach to overcome such constraints [55,56,57].

## 3. Plant Tissue Culture in *Agave* Species

Plant tissue culture is a tool in plant biotechnology. Its applications range from basic research to commercial production [12]. Experimentally, in vitro culture has enabled detailed investigations into physiological and molecular processes, such as embryogenesis, organogenesis, and the biosynthesis of secondary metabolites [11,58,59]. It also supports the functional validation of genes through overexpression and gene silencing strategies [12]. In *Agave* species, the most relevant techniques are those that enable large-scale propagation, genetic conservation, and physiological studies [60].

From an applied perspective, micropropagation is an efficient method for vegetative multiplication of *Agave* species, which exhibit limited sexual reproduction [61]. In *Agave marmorata* Roezl has reported a sexual germination rate as low as 0.42% [62]. This low efficiency, combined with the overexploitation of wild populations, has severely reduced the availability of these species in their natural habitats. These factors highlight the urgent need for conservation strategies [32].

Micropropagation enables the rapid, controlled production of genetically uniform, pathogen-free plants using small portions of plant tissue, known as explants [60]. This process takes place under sterile laboratory conditions and involves several stages, including explant disinfection, induction of adventitious shoots, rooting of the regenerated shoots, and acclimatization of the plants to either a greenhouse or a natural environment, complete plants can be obtained [61]. In addition to organogenesis, somatic embryogenesis provides a complementary pathway for regeneration [59]. Here, somatic embryos are induced from individual cells or callus tissue. These embryos develop structures similar to seedlings and can mature into complete plants under suitable conditions [63].

These approaches have facilitated the development of micropropagation protocols that serve as efficient means for producing healthy plants in limited space with high multiplication rates [60].

Micropropagation protocols enable accelerated propagation and germplasm conservation, thereby facilitating the availability of high-quality plant material for integration into agricultural systems, reforestation strategies, and sustainable production [13].

**Figure 1:**
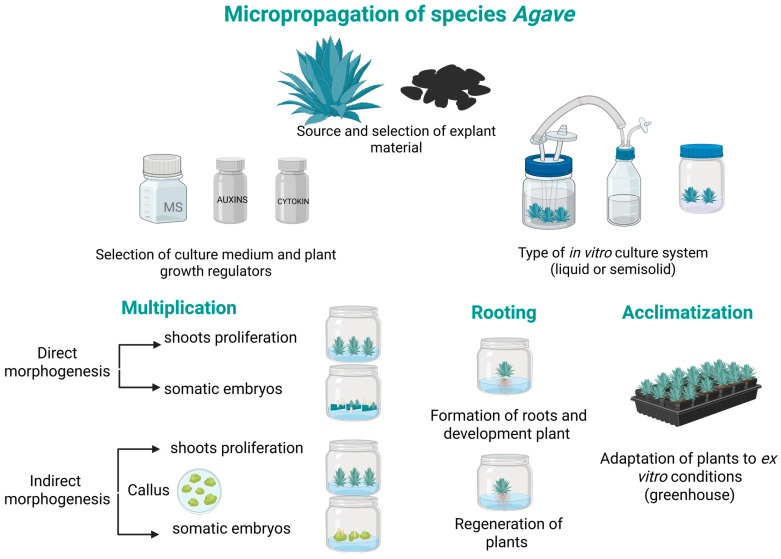
Micropropagation Strategies in *Agave* Species. This figure illustrates the sequential stages involved in the micropropagation of *Agave* species, beginning with explant selection and progressing through culture media formulation with specific PGRs, use of semisolid or liquid systems, morphogenic induction, and final acclimatization to ex vitro [13,14,59].

Figure 1 represents the stages that comprise a typical micropropagation protocol for *Agave* species. It highlights the importance of proper selection of explant sources, formulation of culture medium, and precise choice of PGRs elements that collectively determine the efficiency of the in vitro process.

### 3.1. Applications of PGRs in the In Vitro Culture of Agave Species

Specifically, within the *Agave* genus, PGRs have gained strategic relevance in the design of clonal propagation protocols for developing efficient micropropagation systems that produce healthy plants in limited space with high multiplication rates [15,60].

Among the various categories of PGRs applied in agave tissue culture, cytokinins have received particular attention due to their pivotal role in shoot proliferation [22,64]. Notably, within the *Agave* genus, cytokinins consistently play a crucial role in micropropagation protocols [13]. For agroindustrially relevant species such as *A. marmorata* Roezls and *A. potatorum* Zucc., temporary immersion systems enriched with cytokinins have promoted shoot proliferation, increased chlorophyll content, and reduced hyperhydricity, enhancing plantlet quality and propagation efficiency [65,66,67].

In *A. guiengola* Gentry, BA stimulated both shoot induction and nodular callus formation, with immersion culture systems further amplifying morphogenic responses [55]. Furthermore, in *A. tequilana* Weber, the combined application of BA and kinetin significantly improved shoot multiplication, particularly when integrated with complementary techniques such as stem sagittal sectioning or the addition of auxins [57]. However, in *A. angustifolia* Haw., elevated concentrations of BA have been associated with somaclonal variation, potentially mediated by epigenetic modifications. This suggests a dual role of cytokinins: promoting organogenesis while simultaneously triggering phenotypic variability under stress conditions [68].

More than half of the *Agave* species are endemic to Mexico, many of which are listed under risk categories [32]. *A. peacockii* Croucher, classified as a threatened species requiring special protection, exemplifies this dual application of cytokinins in both propagation and conservation. A study demonstrated that BA combined with kinetin enhances in vitro shoot multiplication while complementing cryopreservation strategies [69]. These results emphasize the fundamental importance of cytokinins as the primary regulators in the in vitro culture of *Agave* species.

The exogenous application of auxins in *Agave* species has been extensively studied in the framework of plant tissue culture. Auxin perception occurs in the cytosol, where it triggers a signaling cascade that culminates in the activation of Auxin Response Factors (ARFs) in the nucleus, thereby promoting the transcription of early regulatory genes [70]. In species such as *A. tequilana* Weber var. azul, *A. angustifolia* Haw., and *A. fourcroydes* Lem., differential morphogenic responses to exogenous auxins have been reported, particularly in response to IAA and 2,4-D. In *A. tequilana* Weber, genomic studies identified 32 ARF genes, with several of them showing differential expression under treatments with specific PGRs, revealing complex regulatory patterns that vary according to both the species and the type of auxin applied [70]. In *Agave* species, root induction has been extensively studied using various auxinic compounds. Among these, indole-3-butyric acid (IBA) and indole-3-acetic acid (IAA), a naturally occurring auxin, have also been employed, although their rapid metabolism in plant tissues often limits their effectiveness [71,72].

Among others, PGRs used in *Agave* species are reported, such as acid abscisic (ABA), which have been shown to modulate somatic embryogenesis and osmotic stress responses in agave tissue culture [73]. In *A. amica* L., 3.78 µM of ABA in combination with maltosa can simulate water stress conditions, promoting the differentiation and maturation of somatic embryos [74]. In various *Agave* species, ABA has been shown to enhance somatic embryo formation, particularly when combined with osmotic agents such as sucrose and polyethylene glycol (PEG), resulting in asynchronous yet successful embryogenesis. Treatments with specific ABA concentrations have been associated with high conversion rates of somatic embryogenesis tissues, achieving up to 100% survival under ex vitro conditions [73].

PGRs, inhibitors, or growth retardants are applied exogenously in vitro culture, especially in strategies aimed at conserving germplasm and maintaining vegetative structures for prolonged periods [75]. Paclobutrazol (PBZ) is a known inhibitor of gibberellin biosynthesis, resulting in significant modifications to plant structural growth [23]. In *A. potatorum* Zucc., the application of PBZ has been shown to preserve shoot viability for up to 180 days under in vitro conditions, without compromising morphogenic integrity [76].

The recognition of nitric oxide (NO) as an endogenous regulator in various physiological processes has led to its increasing application in plant tissue culture over the past decade [22,23]. This application has primarily involved the exogenous addition of sodium nitroprusside (SNP), a widely used NO donor and the most extensively studied member of the iron nitrosyl family [77]. In species of the *Agave* genus, experimental evidence has confirmed its potential as a growth modulator. In *A. angustifolia* Haw., the incorporation of SNP at moderate concentrations (20–40 µM) significantly enhanced shoot proliferation and elongation, whereas higher doses induced phytotoxic effects associated with oxidative stress [78].

In *Agave* species, specific PGRs and osmotic treatments significantly enhanced fructooligosaccharides (FOS) accumulation, linked to the activation of key biosynthetic genes. In *A. tequilana* Weber, salicylic acid (1 mM), abscisic acid (50 mM), sucrose (8%), and kinetin (4.64 µM) promoted up to 36-fold increases in FOS, while in *A. inaequidens* Koch, methyl jasmonate (200 μM) induced the highest response (85-fold). These effects correlated with increased expression of *1-FFT* and *1-SST*, achieving maximum fresh weight in both species [79].

The exogenous application of putrescine has proven to be a promotive agent in the maturation and germination of somatic embryos in *Agave* species. Its incorporation into culture media represents an alternative for optimizing in vitro regeneration protocols, with direct implications for the commercial production of economically important *Agave* species [80].

Numerous investigations have demonstrated that growth regulators such as brassinosteroids, strigolactones, and jasmonates can trigger distinct physiological and morphogenic responses in the in vitro culture of various plant species [81,82,83,84,85,86,87]. Nevertheless, despite extensive research in other plant models, there is a conspicuous absence of studies exploring the application of these growth regulators in the in vitro culture of *Agave* species. This gap presents an opportunity to investigate their potential in this economically and biotechnologically significant genus.

### 3.2. Comparative Overview of Agave In Vitro Protocols

Several previous studies have demonstrated the effectiveness of in vitro methodologies for propagating *Agave* species, positioning this approach as a highly efficient and versatile biotechnological strategy, particularly in the conservation of threatened wild species and the intensive production of economically valuable cultivars [13,17,18].

The implementation of culture systems based on shoot induction from basal meristems has proven successful across a wide range of species, including *A. cupreata* Trel. & A. Berger, *A. karwinskii* Zucc., *A. palmeri* Engelm, *A. potatorum* Zucc., *A. salmiana* Otto ex Salm-Dyck, as well as ornamental species such as *A. victoria-reginae* T. Moore and *A. titanota* Gentry [17]. The morphogenic response of explants is strongly influenced by the type and concentration of cytokinins incorporated into the culture medium, with benzyladenine (BA), 6-γ, γ-dimethylallylaminopurine (2iP), kinetin (Kin), thidiazuron (TDZ), and metatopolin (MT) being among the most commonly employed [17,18]. However, the wide variability observed in shoot proliferation rates ranging from 2.2 to 30 shoots per explant, depending on the species, underscores the need for species-specific protocol optimization [17].

The physiological and morphogenetic diversity among *Agave* species has led to the development of a wide range of in vitro culture protocols. To synthesize and contrast this information, Table 1 provides a comparative overview of recent studies (2019–2025) conducted on different *Agave* species, highlighting the experimental conditions used, the types and concentrations of PGRs applied, and the observed effects in terms of shoot proliferation, organogenesis, somatic embryogenesis, or conservation.

This review highlights the diversity in the use of PGRs across tissue culture protocols applied to more than 15 species of the *Agave* genus. These methodologies have been predominantly implemented in semisolid Murashige and Skoog (MS) medium, although some studies have incorporated temporary immersion systems (RITA^®^, SETIS™) to enhance multiplication efficiency [54,55]. The application of temporary immersion systems has significantly improved multiplication rates and reduced hyperhydricity, as observed in species such as *A. marmorata* Roezl and *A. potatorum* Zucc. [62,67]. Recent studies have revealed that *A. tequilana* Weber, *A. angustifolia* Haw., *A. potatorum* Zucc., and *A. marmorata* Roezl are the most thoroughly investigated species, reflecting their economic and ecological significance. However, lesser studied species such as *A. wocomahi* Gentry and *A. maximiliana* Baker also exhibited improved responses, suggesting potential for conservation and propagation initiatives [96,104].

The integration of novel factors, such as nanoparticles or temporal immersion systems, introduces modifications to the use of different concentrations of PGRs in relation to their effects or responses on various *Agave* species, opening opportunities for continued exploration of the protocols modified by specific parameters for each species [101].

Somatic embryogenesis has been successfully induced in several *Agave* species, including *A. tequilana* Weber, *A. americana* L., *A. cupreata* Trel. & A. Berger, *A. salmiana* ex Salm-Dyck, *A. rzedowskiana* Gentry, *A. wocomahi* Gentry, and *A. amica* L. The most commonly used PGRs in these protocols are 2,4-dichlorophenoxyacetic acid (2,4-D) and benzyladenine (BA), applied at varying concentrations depending on the explant type and culture medium. These compounds promote the induction of embryogenic calli with distinctive morphological traits, such as friable texture and nodular coloration, which are indicative of embryogenic potential. Somatic embryo formation rates range from 4 to 42 embryos per explant, demonstrating high efficiency under specific conditions. Additionally, the application of abscisic acid (ABA) and putrescine (Put) is particularly effective in promoting embryo maturation [73,80]. In *A. tequilana* Weber, the use of picloram (PIC) and BA enabled the production of over 50 somatic embryos per explant, demonstrating the effectiveness of specific combinations in embryogenic induction [99].

Organogenesis, both direct and indirect, has also been extensively documented in species such as *A. angustifolia* Haw., *A. marmorata* Roezl, *A. potatorum* Zucc., *A. peacockii* Croucher, *A. salmiana* ex Salm-Dyck, and *A. tequilana* Weber. The most frequently used PGRs include BA, indole-3-acetic acid (IAA), and 2,4-D. Under optimal conditions—particularly in temporary immersion systems like RITA^®^ up to 81 shoots per explant have been obtained, reflecting a high regenerative capacity. The efficiency of organogenesis is influenced by several factors, including the type of explant, the nutritional status of the mother plant, and the synergistic interaction between growth regulators. These findings emphasize the importance of optimizing physiological and environmental conditions to maximize morphogenic responses in *Agave* species.

Among the most frequently employed PGRs are BA, 2,4-D, IAA, and IBA, used in various combinations and concentrations. BA emerges as the most versatile regulator, utilized alone or in combination. In *A. peacockii* Croucher, the combination of BA and KIN resulted in up to 87 shoots per explant [69], whereas in *A. americana* L., protocols involving BA and 2,4-D were ineffective for shoot multiplication [88].

Quantitative variability across studies is considerable. On *A. marmorata* Roezl, up to 23 shoots per explant were obtained using semisolid MS medium (25%), while *A. duranguensis* Gentry yields only 1.4 shoots in semisolid medium MS, with a similar concentration of BA, suggesting significant genotypic and methodological influence between species [56,76].

A critical aspect that remains underexplored in most studies is the genetic stability of shoots. Some reports, such as those involving *A. angustifolia* Haw., document somaclonal variation and DNA methylation changes associated with high BA concentrations, highlighting the need to incorporate molecular analyses into large-scale propagation protocols [68].

## 4. Conclusions and Future Perspectives

The in vitro culture of *Agave* species represents an effective strategy to overcome the biological and agronomic limitations associated with conventional propagation methods, while also addressing the growing demand for plants intended for productive use, germplasm conservation, and the reinforcement of reforestation programs. In this context, PGRs have been essential for the development of efficient micropropagation protocols, enabling the induction of specific morphogenic responses such as organogenesis, somatic embryogenesis, and rooting. Cytokinins (BA, BAP) and auxins (2,4-D, IAA, IBA) have been the most commonly used and extensively optimized compounds, demonstrating their effectiveness in regenerating complete plants from various explant types.

The reviewed studies highlight that the strategic application of these PGRs has significantly contributed to the research and production of high-quality plant material. However, considerable variability in morphogenic responses among *Agave* species has been observed, indicating that protocols must be specifically tailored according to genotype, explant type, and physiological conditions. This diversity opens new perspectives for exploring alternative PGRs and novel combinations of classical and emerging compounds to improve protocol efficiency and expand the biotechnological potential of the *Agave* genus within a framework of sustainability and integrated utilization.

## Figures and Tables

**Table 1:** Comparative overview of Agave in vitro protocols and PGRs applications (2019–2025).

Species	Plant Tissue Culture	PGRs	Concentration (µM)	Culture Conditions	Effect	Reference
*A. americana* L.	Shoot induction	2,4-D BA	0.09 44.0	Semisolid MS medium reduced in KNO_3_ and NH_4_NO_3_	Although shoot formation occurred on the explant surface, the protocol appeared inadequate for supporting further growth and multiplication.	[88]
	Multiplication	BA NAA	13.2 0.54	Semisolid MS medium with vitamins L2	A significantly higher multiplication rate was achieved from a single explant.	
	Direct somatic embryogenesis	PIC	10.25	Semisolid MS medium with vitamins L2	Somatic embryo formation was induced in 66.7% of the explants.	
	Indirect somatic embryogenesis	Dicamba	6.75	Semisolid MS medium with vitamins L2	The observed embryogenic callus was friable, consisting of small, creamy, globular, and elongated cells that subsequently developed into somatic embryos.	
*A. amica* L.	Callus induction	2,4-D NAA	2.25 2.7	Semisolid MS medium	The treatment led to a 100% callus-induction rate, the highest observed.	[74]
	Callus multiplication	BAP 2,4-D	4.4 4.5	Semisolid MS medium	The callus diameter doubled after two subcultures and increased fivefold by approximately 120 days.	
	Initiation of embryogenic callus	2,4-D	4.5	Semisolid MS medium	The treatment resulted in the formation of nodular, friable, and semi-compact embryogenic callus after 90 days.	
	Development of somatic embryos	ABA	3.8	Semisolid MS medium 45 g L^−1^ maltose	Globular embryoids with proper formation initiated greening and root–shoot axis differentiation.	
	Rooting and bulb formation	IBA	3.67	Semisolid MS medium	Bulb formation occurred at the shoot base, culminating in the development of roots.	
*A. angustifolia* Haw.	Organogenesis and assessment of somaclonal variation	BA	88.8	High BA concentration in culture media (5 months)	Enhanced organogenic capability, increased phenotypic variation, and changes in DNA methylation levels.	[68]
	Indirect organogenesis and evaluation of explant origin (mother plant)	BAP IAA	17.7 5.7	Semisolid MS medium supplemented with coconut water Explants derived from mother plants subjected to fertigation treatments	The combination of growth regulators correlated with the nutritional status of the mother plant under 100% fertigation, resulting in up to 32.7 shoots per explant in vitro culture.	[89]
	Callus induction from embryonic axes	2,4-D BAP	23.0 13.0	Semisolid MS medium with vitamins L2	Callus with an average weight of 0.2 g, suitable for somatic embryo maturation.	[80]
	Somatic embryo maturation	Put	1700	Semisolid MS medium (60 days of incubation)	Exogenous putrescine increased the number of somatic embryos to 21.2 per explant.	
	Somatic embryo maturation	ABA	34.2	Semisolid MS medium (50%)	ABA-induced stress promoted the maturation of somatic embryos, enabling them to regenerate plantlets.	[73]
	Callus induction from embryonic axes	2,4-D BA	22.6 13.3	Semisolid MS medium with L2 vitamins	Dedifferentiation into callus with embryogenic features, friable and beige.	
	Organogenesis stem tissue	BA IAA	4.44 1.71	Semisolid MS medium	In 4 explants, organogenesis response: adventitious buds formed.	[90]
*A. angustifolia* Haw. “Bacanora”	Inoculum evaluation and multiplication	BA 2,4-D	44.4 0.10	Modified MS medium, Temporary Immersion System Frequency: 1 min/6 h	Growth regulators and inoculum density of 20 explants promoted the highest shoot formation (3.05 vigorous shoots) in TIS.	[91]
*A. cupreata* Trel. & A. Berger	Induction of embryogenic callus	2,4-D BA	23.0 13.0	Semisolid MS medium with vitamins L2	The percentage of embryogenic calluses was 66.67 ± 0.48%.	[80]
	Maturation of somatic embryos	Put	1700	Semisolid MS medium	The mean number of somatic embryos per explant was 38.80 ± 9.88.	
	Induction of embryogenic callus	2,4-D BA	22.5 13.2	Semisolid MS medium (75%)	Both non-embryogenic calluses (compact, whitish, and smooth) and embryogenic calluses (friable, beige) were produced, with embryogenic callus accounting for 33.30 ± 14.64%.	[73]
	Maturation of somatic embryos	ABA	34.2	Semisolid MS medium (50%)	Somatic embryo formation was observed at 90 days, with an average of 4.80 ± 3.62.	
	Induction of pro-embryogenic calluses	2,4-D	4.05	Semisolid MS medium (25%) with vitamins L2	Efficient formation of proembryogenic masses by 90%.	[92]
	Expression of direct somatic embryos	IAA	2.9	Semisolid MS medium (50%)	The concentration promoted the efficient formation of 7 direct somatic embryos per explant.	
*A. duranguensis* Gentry	Multiplication	BA	17.4	Semisolid MS medium	A higher average number of shoots, 1.40 per explant, and a higher number of leaves per shoot, 2.70, were obtained.	[56]
*A. guenguiola* Gentry	Multiplication	BA	8.8	Semisolid MS medium	An average of 3.70 shoots per explant was obtained and shoot clusters were successfully generated.	[55]
	Multiplication	BA	4.4	MS medium, Temporary immersion system	A propagation rate of 43 shoots per shoot cluster was achieved.	
*A. marmorata* Roelz	In vitro establishment	BAP	13.2	Semisolid MS medium with ascorbic acid and cysteine	After 30 days of cultivation, the tips were transferred to the multiplication phase.	[66]
	Shoot Multiplication	BAP IAA	13.2 17.2	Temporary immersion system (2 min / 8 h)	For the variable number of shoots per explant, the highest multiplication rate was observed in temporary immersion, with 19.60 shoots and an average size of 1.74 cm, and the lowest percentage of hyperhydricity (3.33%).	
	Multiplication	BAP IAA	13.2 11.4	Semisolid and liquid MS medium in the SETIS™ Frequency of 2 min/8 h for 45 to 60 days	Efficient multiplication of shoot clusters was obtained.	[62]
	Acclimatation	BAP IAA	13.2 5.7	Semisolid MS medium	Efficient shoot multiplication was obtained. Plants were obtained for mycorrhizal fungi treatments.	[93]
	Direct organogenesis	BAP IAA	44.4 57.0	Semisolid MS medium	IAA increased shoot and root length; BA + IAA promoted shoot proliferation, yielding up to 41 shoots per explant and 100% survival rate during acclimatization.	[94]
	Induction of callus	BA 2,4-D	13.3 22.6	Semisolid MS medium (25%) with vitamins L2	Formation of callus masses that had a diameter of 10 and 20 mm with a weight of 0.5 g.	[95]
	Maturation of somatic embryos	2,4-D BA (Pretreatment)	0.45 44.4	Semisolid MS medium (50%)	19.4 somatic embryos were obtained per explant.	
	Maturation of somatic embryos	GA_3_ BA	8.6 44.4	Semisolid MS medium (50%)	Pretreatment with 44.40µM of BA formed 15.2 somatic embryos.	
	Callus induction	BA 2,4-D	0.44 0.45	Semisolid MS medium (25%) with vitamins L2 and MS	Obtaining compact and yellowish calluses, it was observed that the higher the concentration of auxin, the greater the weight of the callus.	[76]
	Shoot induction via indirect organogenesis.	BA	22.2	Semisolid MS medium (25%) with activated carbon	The highest number of shoots regenerated was 24.7 per explant.	
	Shoot induction via direct organogénesis	BA	22.2	Semisolid MS medium (25%) with activated carbon	22.3 shoots were obtained per explant from the meristematic zone explant.	
	Organogenesis stem tissue	BA IAA	4.4 1.7	Semisolid MS medium	Organogenesis response, in 8 explants, the formation of adventitious buds occurred.	
*A. maximiliana* Baker	Axillary multiplication	BA 2,4-D	8.8 0.09	Semisolid MS medium	A total of 26.93 new shoots were produced, exhibiting typical morphological quality.	[96]
*A. nussaviorum* García-Mendoza	Rooting	IBA	2.5	Semisolid MS medium (60%)	The highest percentage of shoots with roots was 83%.	[72]
*A. peacockii Croucher*	Multiplication	BA KIN	26.6 27.8	Semisolid MS medium	The combination significantly favored the morphogenetic response and produced the highest shoot generation with 87 shoots on average.	[62]
*A. potatorum* Zucc.	Rooting	IBA	29.2	Semisolid MS medium reduced in NH_4_NO_3_	A significantly higher number (8.60 ± 1.01) of formed roots was observed compared with the control treatments.	[75]
	Multiplication	BA	8.8	Semisolid MS medium	The significant treatment produced 6.60 shoots on average, with an average length of 4.53 cm.	[67]
	Multiplication	BA	8.8	Medium MS in System temporary immersion RITA^®^	An average of 14.4 shoots was obtained with an average of 2.3 cm.	
	Direct organogenesis	BAP IAA	13.3 17.2	Semisolid MS medium	Regeneration of adventitious shoots 9.73 shoots per explant.	[97]
	Rooting	IAA	17.1	Semisolid MS medium	Improved shoot development was achieved, with an average length of 5.77 cm.	
	Rooting	IBA	2.85	Semisolid MS medium (75%)	96% of shoots developed roots and stems (6.4 mm diameter).	[98]
	Rooting Acclimatization	IBA	2.85 or 5.70	Semisolid MS medium	The addition of IBA to the culture medium enhanced plant growth, resulting in wider leaves, greater stem diameter, higher dry biomass, and overall larger plant size compared with shoots cultured without IBA.	[71]
*A. potatorum* var. “Tóbala”	Direct Organogenesis	BAP 2,4-D	6.6 2.2	Semisolid MS medium (75%) Citric/ascorbic acid	12.5 shoots/stem and leaf explant. More than 70% of the plants survived in the greenhouse after two months of cultivation.	[65]
	Indirect Organogenesis	BAP 2,4-D	8.8 2.2	Semisolid MS medium (50%) Citric/ascorbic acid	Stem explants yielded up to 81 shoots, demonstrating high propagation efficiency.	
*A. rzedowskiana* Gentry	Regeneration by somatic embryogenesis	2,4-D BA	9.0 2.6	Reduced semisolid MS medium in NH_4_NO_3_ Nopal flour 2 g/L^−1^	Direct formation of embryogenic structures at the early scutellar, initial multicellular, and coleoptilar stages.	[99]
*A. salmiana* ex Salm-Dyck	Expression and maturation of somatic embryos	ABA	34.2	Semisolid MS medium (50%)	Mean embryos per explant: 15.4 ± 3.62.	[73]
	Induction of embryogenic callus	2,4-D BA	0.45 0.44	Semisolid MS medium (25%)	It promoted callus formation, resulting in mucilaginous callus.	[75]
	Shoot induction via indirect organogenesis	BA	44.0	Semisolid MS medium (25%) with activated carbon	The highest number of shoots obtained was 23.80 per explant.	
*A. salmiana* Otto ex Salm-Dyck subsp. salmiana	Induction of embryogenic callus	2,4-D BAP	9.0 1.3	Semisolid MS medium with vitamins L2	Statistically significant treatment in quantifying the number of calluses expressing somatic embryogenesis.	[100]
	Expression of somatic embryos	BAP 2,4-D	0.40 4.50	Semisolid MS medium	A maximum of 42.41 ± 5.85 somatic embryos were generated.	
*A. salmiana* Otto ex Salm-Dyck var. “ayoteco”	Axillary multiplication	2,4-D BAP	0.078 57.46	Semisolid MS medium 2250 mg/L zinc nanoparticles	Differentiated shoot development, including leaf formation, was observed at 60 days.	[101]
*A. sisalana* Perr.	Axillary shoot proliferation	TDZ	4.5	Semisolid MS medium	The optimum significant shoot proliferation (14.67 shoots/explant).	[102]
*A. tequilana* Weber	Regeneration by somatic embryogenesis	PIC	2.1	Reduced semisolid MS medium in NH_4_NO_3_ Nopal flour 2 g/L	The treatment was effective in generating good-quality calluses and proembryogenic structures at all stages in leaf explants.	[99]
*A. tequilana* Weber cv. “chato”	Indirect somatic embryogenesis	PIC BAP	49.6 3.32	Semisolid MS medium	The highest average number of somatic embryos was produced (52.43 ± 5.74)	[103]
*A. tequila* Weber var. “azul”	Axillary multiplication Segmented stem explants	BA KIN	13.2 18.8	Semisolid MS medium	BA and KIN increased shoot number per explant, up to 18 (BA) and 26 (KIN); sagittal segmentation also increased axillary budding.	[57]
	Axillary multiplication Segmented stem explants	BA IAA	13.2 5.7	MS liquid medium System temporary immersion RITA^®^ Frequency of 5 min/4 h	The highest IAA concentration resulted in 20 shoots per explant.	
*A. wocomahi* Gentry	Multiplication	BA	4.4	Semisolid MS medium	Resulted in the generation of 11.70 ± 4.8 shoots per explant.	[104]
	Callus tissue induction	PIC BA	6.1 17.6	Semisolid MS medium	A greater callus induction rate (99.16%) was achieved in stem explants, with nodular callus tissue prevailing.	
	Somatic embryo induction	BA	13.2	Semisolid MS medium	A greater number of somatic embryolike structures were obtained.	

## Data Availability

No new data were created or analyzed in this study. Data sharing is not applicable to this article.

## Abbreviations

The following abbreviations are used in this manuscript:

PGRs	Plant growth regulators
IAA	Indole acid acetic
IBA	Indole-3-Butyric acid
NAA	Naphthaleneacetic acid
2,4-D	2,4-Dichlorophenoxyacetic acid
BA	Benzyladenine
BAP	6-Benzylaminopurine
KIN	Kinetin
TDZ	Thidiazuron
GA	Gibberellins
GA_3_	Gibberellic acid
ABA	Abscisic acid
PBZ	Paclobutrazol
SA	Salicylic acid
JA	Jasmonic acid
MeJA	Methyl jasmonate
BRs	Brassinosteroids
NO	Nitric Oxide
SNP	Sodium nitroprusside
SLs	Strigolactones
SE	Somatic embryogenesis
MS	Murashige and Skoog medium
PEG	Polyethylene glycol
FOS	Fructooligosaccharides
ARR	Arabidopsis Response Factors
ARF	Auxin Response Factors
TIS	Temporary Immersion System
RITA^®^	Automated Temporary Immersion Bioreactor
SETIS^®^	Static Temporary Immersion Bioreactor
